# Dose-Response Relationship Between Physical Activity and the Morbidity and Mortality of Cardiovascular Disease Among Individuals With Diabetes: Meta-Analysis of Prospective Cohort Studies

**DOI:** 10.2196/54318

**Published:** 2024-08-19

**Authors:** Yang Chen, Xingsheng Jin, Guochong Chen, Ru Wang, Haili Tian

**Affiliations:** 1 School of Exercise and Health Shanghai University of Sport Shanghai China; 2 Department of Nutrition and Food Hygiene School of Public Health Soochow University Suzhou China

**Keywords:** cardiovascular risk, diabetics, exercise, dose-response association, meta-analysis, physical activity, diabetes

## Abstract

**Background:**

Diabetes, a chronic condition affecting various organs, is frequently associated with abnormal lipid metabolism, notably increased cholesterol and triglyceride levels. These lipid abnormalities are closely linked to the development and advancement of cardiovascular disease (CVD). Although regular physical activity (PA) has consistently shown benefits in reducing CVD risk in the general population, its precise influence on CVD risk among patients with diabetes remains uncertain, particularly regarding dose-response relationships.

**Objective:**

This study aimed to summarize the evidence from prospective studies on the association between PA and CVD morbidity and mortality in individuals with diabetes and explore the optimal levels for public health recommendation.

**Methods:**

We systematically reviewed prospective cohort studies in PubMed, Embase, and Web of Science up to December 2022, with inclusion criteria specifying the studies published in English and included adult participants diagnosed with diabetes. A random effects model was used to pool the relative risk (RR) with the corresponding 95% CI comparing the highest with the lowest PA categories in each study for qualitative evaluation. In addition, linear and spline regression analyses were used to estimate dose-response associations.

**Results:**

The meta-analysis included 12 prospective cohort studies, involving a total of 109,820 participants with diabetes. The combined results revealed that higher levels of PA were associated with a reduced risk of CVD. The RR of CVD for the highest compared with the lowest PA category was 0.62 (95% CI 0.51-0.73). In addition, there were 4 studies describing leisure-time PA, and the pooled RR was 0.68 (95% CI 0.52-0.83) for the highest versus the lowest activity. The linear regression model revealed that each 10 MET (metabolic equivalent of task)-hours per week of incrementally higher PA was associated with a 19% (95% CI 11.6-25.7) and a 6.9% (95% CI 4.5-9.3) reduction in CVD morbidity and mortality. Additionally, spline regression curves showed nonlinear relationships between PA levels and the risk of CVD and CVD mortality (both *P*_nonlinearity_<.001), with a limited reduction in CVD risk and some further reduction in CVD mortality above 20 MET-hours per week of PA levels.

**Conclusions:**

For patients with diabetes, especially type 2 diabetes, there was a dose-response relationship between increased PA and reduced risk of CVD morbidity and mortality. The observed PA threshold is consistent with the recommended level for the general population. Gradually moving from inactivity to a guideline-recommended PA level could therefore significantly reduce the burden of CVD in patients with diabetes.

## Introduction

Diabetes, as a chronic disease, poses a significant risk to the functionality of multiple organs. Alongside the impairment of nerves and blood vessels, diabetes can lead to various severe complications, including retinopathy, nephropathy, and diabetic foot ulcers [1-3]. Data from the International Diabetes Federation showed that 6.7 million people died from diabetes in 2019, and 1 in 10 adults aged 20-79 years had diabetes, totaling 537 million people [4]. Medical spending on diabetes accounts for 9% of the global health spending, totaling US $966 million. By 2045, there will be an increase of 3.6 million people with diabetes worldwide, and the increase in diabetes-related health expenditures will exceed 206 billion, and the number of people who will die from diabetes will increase by 2.5 million [5,6]. Patients with diabetes often exhibit abnormal lipid metabolism, including elevated levels of cholesterol and triglycerides [7]. These abnormal lipid levels are closely associated with the occurrence and progression of cardiovascular disease (CVD) [8]. CVD is a group of diseases caused by atherosclerosis, characterized by lipid deposition and plaque formation on the arterial walls, ultimately leading to vascular narrowing and obstruction [9]. In patients with diabetes, abnormal lipid metabolism accelerates the progression of atherosclerosis, thereby increasing the risk of cardiovascular events such as myocardial infarction and stroke [10]. According to the latest guidelines, adults with diabetes face 2-3 times the risk of developing CVD [11]. The risk of atrial fibrillation in patients with diabetes increases by 3%, leading to higher possibilities of stroke, heart failure, and mortality [11]. CVD remains a leading cause of death worldwide, resulting in approximately 20.5 million fatalities and accounting for one-third of the total global mortality [12,13].

The beneficial role of regular physical activity (PA) in reducing the risk of CVD has been repeatedly confirmed in the general population [14-17]. Through PA, patients with diabetes can gradually establish a healthy lifestyle, thereby improving overall health and reducing the risk of chronic diseases [18]. Regular PA has the potential to enhance insulin sensitivity, improve vascular responsiveness, and optimize cardiorespiratory fitness levels [19-21]. These physiological adaptations may contribute to a reduced incidence of CVD among individuals living with diabetes [22]. The health benefits of PA in individuals with diabetes are mentioned in existing guidelines and public health recommendations. For example, the European Society of Cardiology guidelines for PA in patients with cardiac conditions mentioned the benefits of aerobic exercise and strength training for patients with diabetes for blood glucose and blood pressure control, weight loss and improved exercise capacity, as well as increased exercise capacity and reduced risk of CVD [23]. The World Health Organization [24] recommends that 150-300 minutes of moderate to vigorous PA per week is also applicable to adults with chronic diseases such as diabetes.

However, previous prospective studies have yielded inconsistent results regarding the relationship between PA levels and CVD risk in patients with diabetes. Some literature highlights the health benefits of moderate to high PA levels in reducing CVD risk, particularly at the moderate level [5,25]. Conversely, another study suggests that the association between changes in PA levels and CVD risk factors among patients with diabetes is relatively weak, implying a limited direct impact on cardiovascular health [26]. Consequently, the dose-response relationship between PA and CVD risk remains unclear, requiring additional research for informed public health recommendations.

The primary objective of this meta-analysis was to synthesize evidence from prospective studies to elucidate the relationship between PA and CVD in patients with diabetes. Additionally, we aimed to quantify the weekly metabolic equivalent of task (MET) for PA exposure and clarify the dose-response relationship. This study aimed to provide a theoretical foundation for future exercise prescriptions for individuals with diabetes, ultimately improving health outcomes in this patient population.

## Methods

### Ethical Considerations

This review paper presents a secondary analysis of existing data from previously published original studies rather than a direct collection of new data. As such, ethics approval was not necessary.

### Search Strategy

Electronic literature searches were conducted for cohort studies investigating the association between PA and CVD risk among individuals with diabetes. The searches were performed from inception to December 2022 for relevant studies published in MEDLINE, Embase, and the Web of Science. The study keywords used in the searches were thesaurus terms registered in MEDLINE (MeSH) or Embase (EMTREE), as well as entry words related to diabetes, PA, CVDs, and cohort study.

### Inclusion and Exclusion Criteria

The inclusion and exclusion criteria for the study are shown in Textbox 1.

**The inclusion and exclusion criteria for the study.**

**Inclusion criteria**

- Paper type: prospective cohort study.
- Population: adult patients with diabetes.
- Disease: outcomes are cardiovascular diseases, including fatal and nonfatal cardiovascular events.
- Methods: studies that provide or allow for the calculation of effect size (ie, relative risk, hazard ratio, or odds ratio) and corresponding SE for high physical activity (PA) categories compared with the lowest PA category.
- Language: studies in the English language.

**Exclusion criteria**

- Paper type: presence of additional nonpharmacological interventions.
- Methods: studies lacking effect sizes or where calculation is not feasible. No stratified comparison of PA.
- Language: languages other than English.

### Selection Process

The titles and abstracts of a large number of publications were obtained using the aforementioned search strategy. These papers were divided equally between the 2 authors (YC and XJ) and initially screened based on the titles. Subsequently, the third author (HT) cross-checked 1100 (15%) of the 7334 documents to ensure accuracy for initial inclusion. The abstracts of the initially included studies were then read independently by both authors (YC and XJ) for inclusion. Any disagreements were referred to the third author (GC) and resolved through discussion. Finally, 1 author (RW) reviewed the full papers, and the second author (HT) cross-checked the included literature to determine the final inclusion criteria.

### Data Extraction

The 2 authors (YC and XJ) independently extracted the key characteristics of the included studies, and discrepancies were resolved through discussion. When multiple effect measures, such as unadjusted and adjusted measures, were present in the included studies, the most fully adjusted measures were selected. The key characteristics of the research included, but were not limited to, the author, year of publication, study population, person-years, follow-up time, cohort status, disease diagnosis methods, and methods of measuring PA.

### Assessment of Study Quality

A quality criteria scale was developed at the study level using applicable elements from the Newcastle-Ottawa scale for cohort studies. This scale has been widely used in meta-analyses of exercises and health risks. The study-level quality assessment was conducted by 2 authors (HT and RW).

### Data Analysis

To assess the qualitative association between PA and CVD risk, we pooled the log relative risk (RR) of the highest versus the lowest PA categories from each study using the inverse variance method. Heterogeneity among the studies was evaluated using *Q* statistics and *I*^2^, both overall and within each stratum after stratification [27]. If significant between-study heterogeneity was observed, a random effects model was used to calculate the pooled estimate [28].

We also conducted subgroup analyses to explore differences between subgroups to search for possible effect modifiers or sources of heterogeneity. To verify possible sources of heterogeneity, stratified analyses were conducted on the following study characteristics that we identified based on previously extracted data from the included studies: mean follow-up duration (≤10 years or >10 years), type of diabetes (1 or 2), diabetes duration (not available, ≤10 years or >10 years), mean age (<60 years or ≥60 years), PA type (total PA or leisure-time PA), the proportion of men (≤50% or >50%), validation of PA questionnaire (no or yes), area (Asia or Europe or North America or mixtures), and mean BMI (≤25, 25, or ≤30 kg/m^2^) [29]. Meta-regression analysis was used to test differences between these strata.

Publication bias was primarily detected by visual assessment using funnel plots in which SE was plotted against log RR for the highest and lowest PA categories in each study. Symmetry in the plot was assumed to indicate no publication bias. In addition, a statistical assessment using the Egger regression asymmetry test confirmed the symmetry [30]. To assess the robustness of our findings, we performed a sensitivity analysis by systematically omitting 1 study at a time to evaluate its impact on the overall pooled results.

We extracted detailed information from the literature that quantified PA in subsequent dose-response analysis studies. If the graded quantification of PA in a paper was not a point estimate, we assumed a consistent width for each category of PA and considered the middle value of its upper and lower limits as the point estimate of PA for this category [31]. The same standard unit (MET-hours) was used to standardize the PA doses reported in the literature.

PA is represented by different forms of exercise, such as walking, running, moderate to vigorous exercise, and sedentary activities. For some studies that did not directly give the corresponding quantitative data, we defined the data according to the compendium, such as for different exercise intensities, using 1.0-1.5, 1.6-2.9, 3-5.9, and ≥6 MET [32]. Following the above expression, we translate it into the corresponding point estimates: 1.5, 4.5, and 7.5 MET. If the literature only stated the average duration of a given exercise, we assumed that the individuals performed it at an intensity of 4.5 MET [33,34].

First, we assumed a log-linear relationship between PA and CVD morbidity and mortality. To investigate this relationship, we used a weighted least squares regression model. In addition, we used restricted cubic spline regression models to further explore the shape of the relationship between PA and CVD outcomes. In both models, we regressed of the log RR for each nonreferent group against a higher dose of PA compared to the lowest PA category. Data analysis was conducted using STATA software (version 17; StataCorp LLC). We considered a 2-sided *P* value of less than .05 to be statistically significant [35].

## Results

### Literature Search

**Figure 1:**
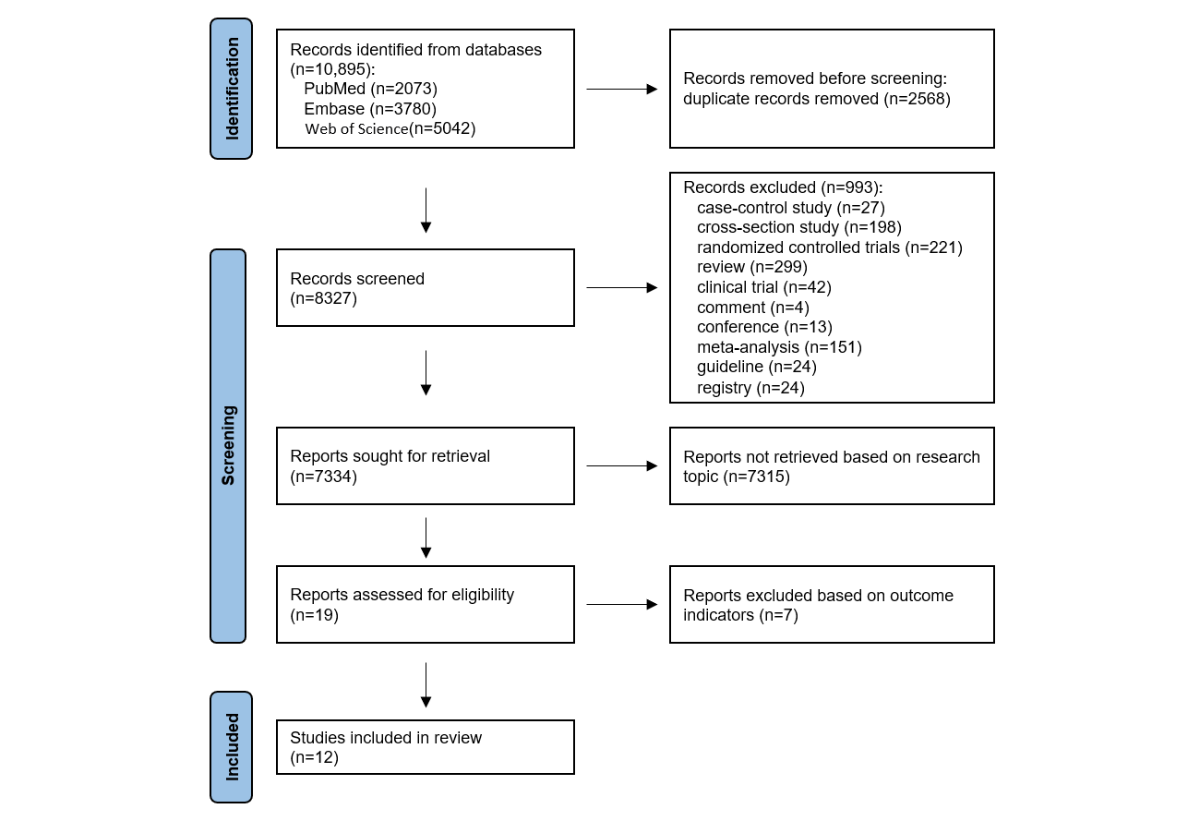
Study selection flowchart according to the PRISMA (Preferred Reporting Items for Systematic Reviews and Meta-Analyses) guidelines.

The complete search process is presented in Figure 1. Table S1 in Multimedia Appendix 1 presents details of the literature search of 8327 papers retrieved from MEDLINE, Embase, and Web of Science electronic literature searches. After a full-text review, 7 of these papers were excluded, with specific details provided in Table S2 in Multimedia Appendix 1 [26,36-41]. A total of 12 studies met the prespecified inclusion criteria [5,42-52]. Table 1 presents the characteristics of the 12 included studies. In total, 5 studies validated the PA questionnaire [43,46,48,49,51]. Most of the included studies were conducted in Europe with a total of 6 studies [44,47-49,51,52]. Of the 12 included studies, only 2 focused on patients with type 1 diabetes [47,48], while the rest exclusively studied patients with type 2 diabetes; 4 studies limited the scope of PA to leisure-time PA [42,46,48,50].

Table S3 in Multimedia Appendix 1 shows details of the confounding factors considered in each included study. In total, 4 studies detailed the patients’ CVD history [5,42,44,51], 7 studies considered social factors [5,42-45,50,51], and 4 studies took dietary factors into account [43,46,50,51]. The consideration of confounders varied among the studies, and more than half of the included studies adjusted the effect measure for all of the 5 following classic CVD risk factors: age, gender, smoking, dyslipidemia, and hypertension. A complete assessment of the quality of the literature is shown in Table S4 in Multimedia Appendix 1.

**Table 1:** Details of characteristics of the included studies, including study region, follow-up duration, duration of diabetes, type of diabetes, and some basic information about patients.

Author (year; area)	Follow-up years, mean (SD)	d_duration^a^ (years), mean (SD)	Age (years), mean (SD)	PA^b^ type (d_type^c^)	PA_verified^d^	BMI (kg/m^2^), mean (SD)	Outcome
Blomster et al (2013; 20+)^e^ [5]	5 (—^f^)	7.9 (6.4)	65.8 (6.4)	Total PA (2)	Yes	28.3 (5.2)	CVD^g^ events
Brown et al (2014; United States) [42]	9.2 (4.9)	10.9 (8.8)	65.5 (—)	Total PA (2)	No	29.51 (—)	CVD mortality
Chen et al (2022; China)^e^ [43]	4.5 (—)	5 (—)	62.9 (—)	Total PA (2)	No	25.29 (—)	CVD mortality or heart disease mortality or stroke mortality
Enguita-Germán et al (2021; Kingdom of Spain) [44]	5 (—)	7.3 (—)	70.5 (—)	Total PA (2)	No	30.6 (—)	CVD events
Inoue et al (2020; United Mexican States)^e^ [45]	8 (—)	— (—)	70.3 (—)	Total PA (2)	No	29.17 (—)	Nonfatal CVD events or fatal CVD event
Sone et al (2013; Japan)^e^ [46]	8.05 (—)	11.0 (7.1)	58.5 (—)	LTPA^h^ (2)	No	23.0 (3.0)	CHD^i^ only, stroke only, or both
Tielemans et al (2013; Europe)^e^ [47]	7 (—)	13.6 (—)	32.7 (10.2)	Total PA (1)	No	23.59 (—)	Total CVD events (men or women)
Tikkanen-Dolenc et al (2017; Finland)^e^ [48]	10.3 (3.4)	21.7 (12.4)	38.8 (12.4)	LTPA (1)	Yes	25.2 (3.6)	CVD events
Vepsäläinen et al (2010; Finland) [49]	18 (—)	7.8 (—)	<60 (—)	Total PA (2)	Yes	29.2 (—)	CVD mortality or CHD mortality
Yen et al (2022; Taiwan [China])^e^ [50]	17 (—)	— (—)	60 (13)	LTPA (2)	Yes	24.7 (—)	CVD mortality
Yerramalla et al (2020; United Kingdom)^e^ [51]	8.8 (6.1)	— (—)	44.9 (6.0)	Total PA (2)	Yes	— (—)	CVD mortality
Zethelius et al (2014; Switzerland)^e^ [52]	4.8 (—)	5.7 (—)	59.9 (—)	Total PA (2)	No	30.04 (—)	Fatal or nonfatal CVD or fatal or nonfatal CHD or fatal CVD

^a^d_duration: duration of diabetes mellitus.

^b^PA: physical activity.

^c^d_type: types of diabetes mellitus.

^d^PA_verified: validation of PA questionnaire.

^e^This study was used for the dose-response analysis between PA and outcomes.

^f^Not available.

^g^CVD: cardiovascular disease.

^h^LTPA: leisure time physical activity.

^i^CHD: coronary artery heart disease.

### Qualitative Assessment of the Association of High PA With CVD Risk

Of the 12 included studies, 5 studies included multiple outcomes, such as coronary artery heart disease (CHD), stroke, and heart disease [43,45-47,49,52]. When the outcomes of one study were fatal or nonfatal CVD, fatal or nonfatal CHD, and fatal CVD, we chose fatal or nonfatal CVD for qualitative analysis because it had greater representation [52]. When the outcomes of the study were CHD or stroke and CHD and stroke, we chose CHD for qualitative analysis [46]. When the outcomes of the study were fatal CVD, heart disease mortality, and stroke mortality, we chose fatal CVD for qualitative analysis [43]. One study included 2 separate outcomes, fatal and nonfatal CVD events, and we combined the estimates using a fixed-effects model [45]. Specific information regarding the studies included in the qualitative analysis is provided in Table S5 in Multimedia Appendix 1.

**Figure 2:**
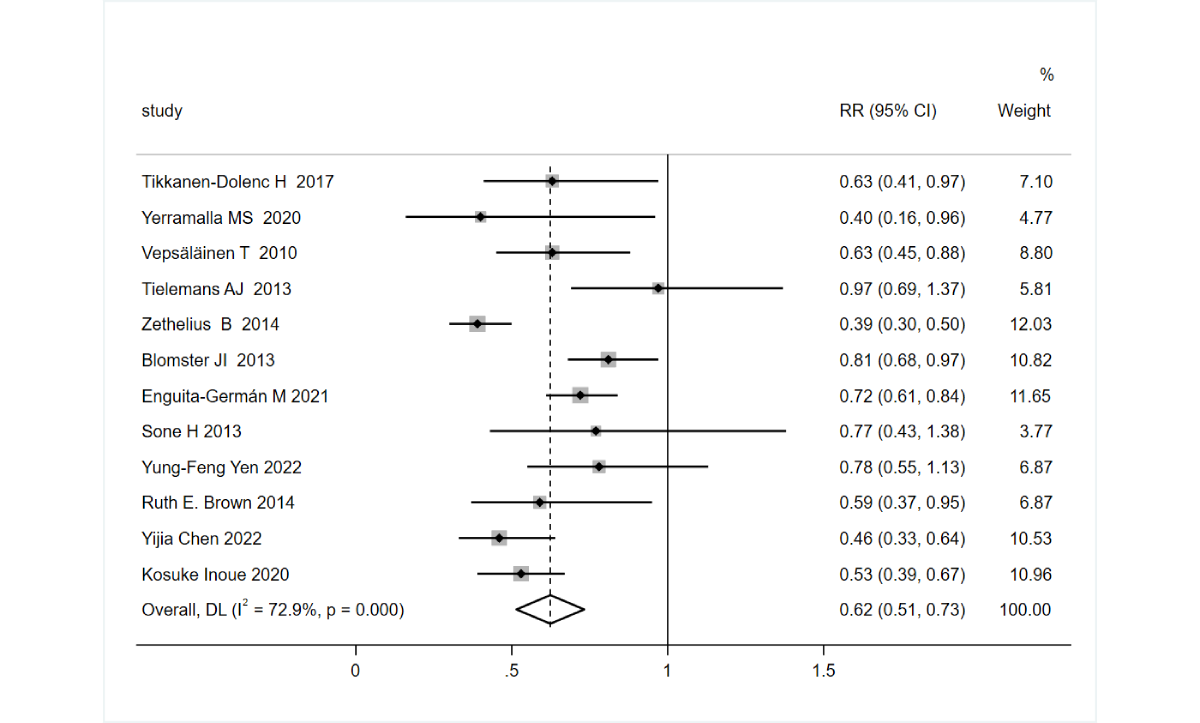
Forest plot illustrating the summary estimate of cardiovascular disease risk for individuals with diabetes in the highest versus lowest physical activity group along with the 95% CI. Study-specific estimates and the overall pooled estimate are depicted by circles and diamonds, respectively. Horizontal lines indicate the range of the 95% CI. The size of the squares represents the weight of each study, with larger squares indicating studies with greater weight [5,36-46]. RR: relative risk.

Figure 2 shows the forest plot for the risk estimate of CVD events in relation to PA in patients with diabetes. As heterogeneity was revealed by the I^2^ statistic (I^2^=72.9%; *P*<.001), a random effects model was used. The pooled RR of the CVD event was 0.62 (95% CI 0.51-0.73).

Table 2 shows the results of the stratified analyses for the key study characteristics in addition to the results of meta-regression analyses testing strata difference. The predefined characteristics did not significantly alter the combined relationship between PA and the risk of CVD. A consistently lower risk of CVD events was observed across all strata.

**Figure 3:**
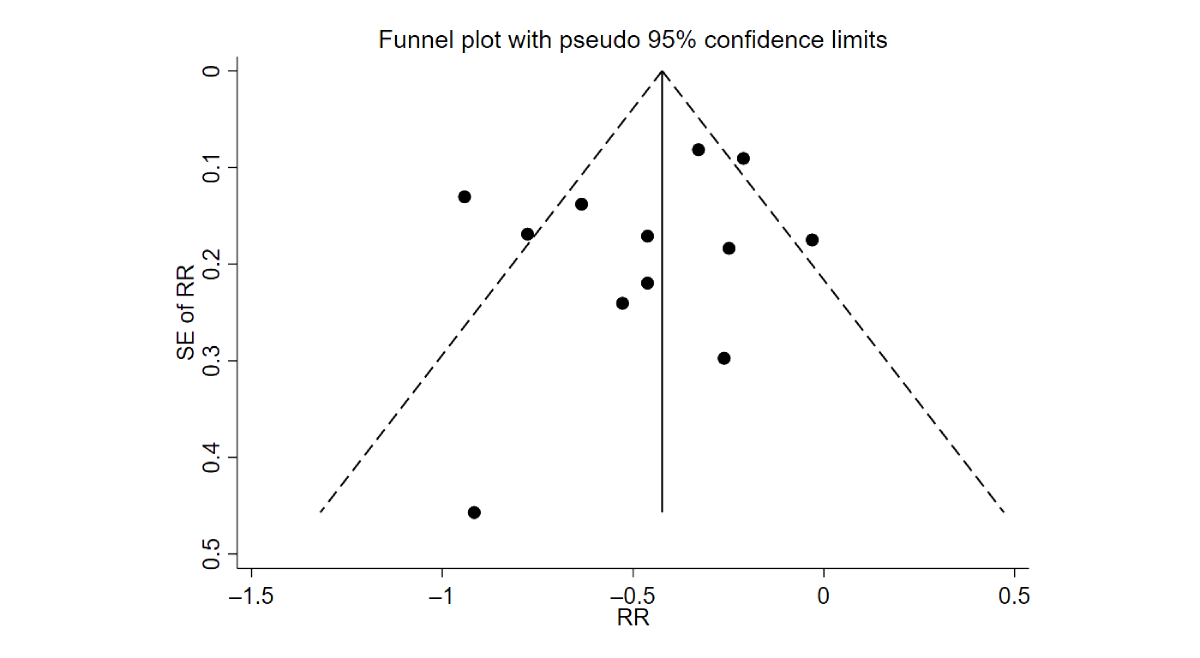
Funnel plot assessing publication bias for the meta-analysis of prospective studies on the relationship between physical activity categories (the highest vs the lowest) and cardiovascular disease risk in individuals with diabetes. RR: relative risk.

To assess publication bias, we visually inspected the funnel plots presented in Figure 3. Both sides of the funnel plot were essentially symmetrical, and the Egger test was used, and there was limited evidence for small-study effects (*t*_X_=1.13; *df*=10; *P*=.29), as shown in Figure S1 in Multimedia Appendix 1. The results of the subgroup analyses did not identify sources of heterogeneity but were consistent for the health benefits of PA, as shown in Figures S2-S10 in Multimedia Appendix 1. The results of the sensitivity analysis consistently supported our main findings, demonstrating the stability and reliability of the results, as shown in Figure S11 in Multimedia Appendix 1.

**Table 2:** Stratified analyses of pooled RR^a^ of cardiovascular disease morbidity comparing high versus low levels of PA^b^.

		Studies, n (%)	RR (95% CI)	*Q* statistics	*I*² (%)	*P* value of heterogeneity	Meta-regression^c^
Total		12	0.62 (0.51-0.73)	40.58	72.90	<.001	—^d^
**Mean follow-up duration (years)**							
	≤10	9 (75)	0.61 (0.48-0.74)	38.43	79.20	<.001	Reference
	>10	3 (25)	0.67 (0.52-0.82)	0.76	0	<.001	0.64
**Type of diabetes**							
	Type 1	2 (17)	0.79 (0.45-1.12)	2.29	56.30	<.001	Reference
	Type 2	10 (83)	0.60 (0.48-0.72)	35.42	74.60	<.001	0.25
**Duration of diabetes (years)**							
	Not to mention	3 (25)	0.57 (0.40-0.75)	3.00	33.40	<.001	Reference
	≤10	5 (42)	0.60 (0.43-0.77)	31.71	87.40	<.001	0.34
	>10	4 (33)	0.71 (0.54-0.89)	3.28	8.50	<.001	0.98
**Mean age (years)**							
	<60	6 (50)	0.60 (0.42-0.79)	15.61	68	<.001	Reference
	≥60	6 (50)	0.64 (0.52-0.77)	16.63	68	<.001	0.77
**PA type**							
	LTPA^e^	4 (33)	0.68 (0.52-0.83)	1.09	0	<.001	Reference
	Total PA	8 (67)	0.61 (0.47-0.74)	38.03	81.60	<.001	0.55
**Men (%)**							
	≤50	6 (50)	0.61 (0.48-0.74)	9.77	48.80	<.001	Reference
	**>**50	6 (50)	0.62 (0.44-0.80)	30.66	83.70	<.001	0.88
**Validation of PA questionnaire**							
	Yes	5 (42)	0.54 (0.43-0.65)	3.46	0	<.001	Reference
	No	7 (58)	0.66 (0.51-0.82)	36.17	83.40	<.001	0.48
**Area**							
	Asia	3 (25)	0.62 (0.38-0.87)	4.54	55.90	<.001	Reference
	Europe	6 (50)	0.61 (0.44-0.79)	25.39	80.30	<.001	0.63
	North America	2 (17)	0.54 (0.42-0.67)	0.13	0	<.001	0.49
	Mixtures	1 (8)	0.81 (0.67-0.95)	0	–	<.001	0.68
**BMI^f^ (kg/m^2^)**							
	<25	2 (17)	0.90 (0.63-1.18)	0.45	0	.50	Reference
	≥25	8 (67)	0.61 (0.50-0.72)	14.61	52.10	.04	0.31
	≥30	2 (17)	0.55 (0.23-0.88)	18.01	94.40	<.001	0.35

^a^RR: relative risk.

^b^PA: physical activity.

^c^Meta-regression: represents test for signiﬁcance of the study modiﬁcation across strata.

^d^Not available.

^e^LTPA: leisure-time physical activity.

^f^BMI: overweight is defined as BMI ≥25 kg/m^2^, and obese is defined as BMI ≥30 kg/m^2^.

### Dose-Response Relationship Between PA and CVD Events and Mortality

**Figure 4:**
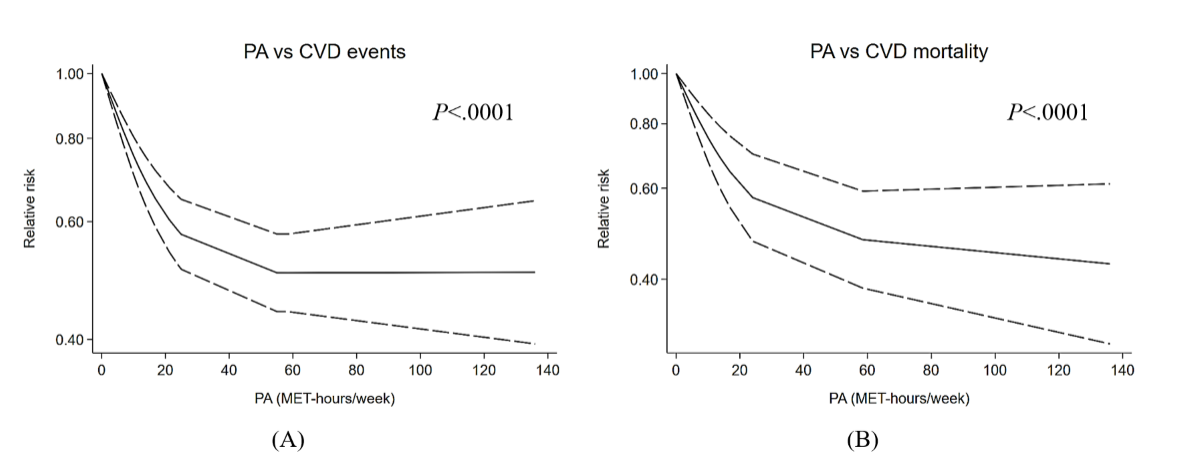
Relationship between weekly PA and relative risk of (A) CVD events and (B) CVD mortality in individuals with diabetes. Spline regression curves depicting the relationship between PA and CVD risk are presented. The solid line represents a log-linear relationship, while the dashed lines indicate the upper and lower limits of the 95% CI. A distinct inflection point is observed at 20 MET-hours of movement per week. The graph exhibits an overall downward trend. CVD: cardiovascular disease; PA: physical activity.

Specific information regarding the studies included in the quantitative analysis is provided in Tables S6-S8 in Multimedia Appendix 1. The log RR of CVD events and mortality against weekly PA in terms of MET-hours in patients with diabetes are described in the linear and spline regression curves (Figure 4). The linear regression model results indicate that a 10 MET-hours per week incrementally higher PA was associated with a 19% (95% CI 11.6-25.7) and a 6.9% (95% CI 4.5-9.3) reduction in total CVD risk and risk of CVD mortality, respectively. The results of the spline regression analysis demonstrated that the relationship between PA and CVD morbidity and mortality was nonlinear (both *P*_nonlinearity_<.001). While there was a limited reduction in the risk of total CVD as PA levels increased, there were additional reductions in the risk of CVD mortality beyond PA levels of 20 MET-hours per week.

## Discussion

### Overview

The global incidence of diabetes, particularly type 2 diabetes, is increasing annually. Patients with diabetes often experience multiple complications, and in severe cases, these complications can cause premature death [53]. Therefore, it is essential to actively guide patients with diabetes toward a healthy lifestyle, which includes regular PA, to prevent cardiovascular complications and other adverse outcomes [54,55]. A healthy lifestyle is inseparable from active exercise, essential for health promotion and well-being enhancement. Exercise plays an important role as a preventive strategy against various chronic diseases, including CVD, stroke, diabetes, osteoporosis, and obesity, and improves the quality of life in terms of mental health [56,57]. It is recommended that individuals with type 2 diabetes engage in regular PA and reduce their sedentary time by taking breaks between sedentary activities.

### Principal Findings

This study aimed to synthesize evidence from prospective studies to elucidate the relationship between PA and CVD risk in patients with diabetes. The main finding of this study revealed a significant inverse correlation between regular PA and the risk of CVD in individuals with diabetes. These results align with previous studies that have shown the positive impact of exercise on glucose levels, β cell function, insulin sensitivity, vascular function, and gut microbiota, contributing to the healthy management of diabetes and reducing the risk of CVD [25].

The results of this study revealed that the trend of decreasing CVD morbidity and mortality slowed after the inflection point and leveled off with increasing PA. It is noteworthy that this slowing trend did not affect the positive effect of PA on CVD risk. Previous studies have suggested that the health effects of high doses of PA are not yet clear and may even be harmful to the cardiovascular system [58]. However, in this study, no negative effects were observed, and any amount of exercise was beneficial in reducing CVD risk for individuals with diabetes.

### Limitations

This study has some limitations. First, in most of the included cohort studies, PA information was mainly obtained using questionnaires, which were differentially applied in different studies, and the findings may have been affected by the potential recall bias. Second, some studies included different end-point outcomes, such as CHD, CHD mortality, and stroke; however, we did not analyze them separately either qualitatively or quantitatively, due to the relatively small number of studies on these CVD subtypes. Third, only a few of the studies included in this review addressed leisure-time PA, and future studies may be needed to further elucidate the effects of specific types of PA on cardiovascular risk in patients with diabetes. Finally, the inclusion of different subtypes of diabetes among the study participants. Type 1 diabetes is typically characterized by autoimmune destruction of pancreatic β cells, resulting from the immune system attacking insulin-producing cells, and is associated with both genetic and environmental factors [59]. In contrast, type 2 diabetes is attributed to insulin resistance and inadequate insulin secretion, closely linked to obesity, unhealthy lifestyles, and genetic factors [60]. Therefore, variations in physiological and pathological profiles between the 2 types may lead to disparate cardiovascular responses to PA. However, according to previous studies, patients with both type 1 and type 2 diabetes benefit from engaging in appropriate exercise routines [57,61-65]. Consequently, we aim to analyze the overall health benefits of PA for all patients with diabetes. Although the FS3 results indicate that different types of diabetes are potential factors causing heterogeneity, they indeed demonstrate health benefits for both types of patients. Given this limitation, we intend to conduct future studies that are more tailored to different subtypes of patients with diabetes as much as possible.

### Comparison to Prior Work

It is worth noting that while previous studies have explored the link between PA and CVD, this study takes a more focused approach to cardiovascular health [66-68]. We conducted thorough analyses, separately examining the risks of CVD and CVD mortality. Additionally, we included relevant research conducted from 2010 to the present, enhancing the timeliness of our findings. Therefore, our research retains significant clinical value.

### Conclusions

This study demonstrated a dose-response relationship between increased PA and decreased risk of CVD morbidity and mortality in patients with diabetes, particularly type 2 diabetes. The observed PA threshold was consistent with the recommended level for the general population, indicating that gradually increasing PA levels from a sedentary state to the recommended guidelines can significantly reduce the burden of CVD in patients with diabetes. These findings support the development and implementation of policies to promote PA among patients with diabetes, particularly those with type 2 diabetes.

## Appendix

Multimedia Appendix 1 Search strategy, study selection, confounders, bias assessment, data summary, subgroup analyses, and sensitivity analysis.

## Abbreviations

CHD: coronary artery heart disease
CVD: cardiovascular disease
MET: metabolic equivalent of task
PA: physical activity
RR: relative risk

## Data Availability

The data sets generated and analyzed during this study are available from the corresponding author on reasonable request.
