# Supplementary material for: Dose-Response Relationship Between Physical Activity and the Morbidity and Mortality of Cardiovascular Disease Among Individuals With Diabetes: Meta-Analysis of Prospective Cohort Studies
Source: JMIR Public Health Surveill. 2024 Aug 19;10:e54318. doi: 10.2196/54318 (PMC11369533; doi:10.2196/54318)
Supplement: Multimedia Appendix 1 [file publichealth_v10i1e54318_app1.docx]

**SUPPLEMENTAL MATERIAL**

Supplementary Table 1. Details of the search strategy for the meta-analysis

EMBASE search

| S1 | [EMTREE terms related to physical activity]  ' physical activity '/exp |
| --- | --- |
| S2 | [entry words related to physical activity]  'Exercise':ab,ti or 'Exercises':ab,ti or 'Activities, Physical':ab,ti or 'Activity, Physical':ab,ti or 'Physical Activities':ab,ti or 'Exercise, Physical':ab,ti or 'Exercises, Physical':ab,ti or 'Physical Exercise':ab,ti or 'Physical Exercises':ab,ti or 'Acute Exercise':ab,ti or 'Acute Exercises':ab,ti or 'Exercise, Acute':ab,ti or 'Exercises, Acute':ab,ti or 'Exercise, Isometric':ab,ti or 'Exercises, Isometric':ab,ti or 'Isometric Exercises':ab,ti or 'Isometric Exercise':ab,ti or 'Exercise, Aerobic':ab,ti or 'Aerobic Exercise':ab,ti or 'Aerobic Exercises':ab,ti or 'Exercises, Aerobic':ab,ti or 'Exercise Training':ab,ti or 'Exercise Trainings':ab,ti or 'Training, Exercise':ab,ti or 'Trainings, Exercise':ab,ti |
| S3 | 1 OR 2 |
| S4 | [EMTREE terms related to diabetes mellitus]  'diabetes mellitus'/exp |
| S5 | [entry words related to diabetes mellitus]  'diabetes':ab,ti |
| S6 | 4 OR 5 |
| S7 | [EMTREE terms related to cardiovascular disease]  'cardiovascular disease'/exp |
| S8 | [entry words related to cardiovascular disease]  'Cardiac Events':ab,ti or 'Cardiac Event':ab,ti or 'Event, Cardiac':ab,ti or 'Adverse Cardiac Event':ab,ti or 'Adverse Cardiac Events':ab,ti or 'Cardiac Event, Adverse':ab,ti or 'Cardiac Events, Adverse':ab,ti or 'Acute Exercises':ab,ti or 'Exercise, Acute':ab,ti or 'Exercises, Acute':ab,ti or 'Exercise, Isometric':ab,ti or 'Exercises, Isometric':ab,ti or 'Isometric Exercises':ab,ti or 'Isometric Exercise':ab,ti or 'Exercise, Aerobic':ab,ti or 'Aerobic Exercise':ab,ti or 'Aerobic Exercises':ab,ti or 'Exercises, Aerobic':ab,ti or 'Exercise Training':ab,ti or 'Exercise Trainings':ab,ti or 'Training, Exercise':ab,ti or 'Trainings, Exercise':ab,ti |
| S9 | 7 OR 8 |
| S10 | [EMTREE terms related to cohort analysis]  'cohort analysis'/exp |
| S11 | [entry words related to cohort analysis]  'Cohort Study':ab,ti or 'Studies, Cohort':ab,ti or 'Study, Cohort':ab,ti or 'Concurrent Studies':ab,ti or 'Studies, Concurrent':ab,ti or 'Concurrent Study':ab,ti or 'Study, Concurrent':ab,ti or 'Closed Cohort Studies':ab,ti or 'Cohort Studies, Closed':ab,ti or 'Closed Cohort Study':ab,ti or 'Cohort Study, Closed':ab,ti or 'Study, Closed Cohort':ab,ti or 'Studies, Closed Cohort':ab,ti or 'Birth Cohort Studies':ab,ti or 'Birth Cohort Study':ab,ti or 'Cohort Studies, Birth':ab,ti or 'Cohort Study, Birth':ab,ti or 'Studies, Birth Cohort':ab,ti or 'Study, Birth Cohort':ab,ti or 'Analysis, Cohort':ab,ti or 'Analyses, Cohort':ab,ti or 'Cohort Analyses':ab,ti or 'Cohort Analysis':ab,ti or 'Historical Cohort Studies':ab,ti or 'Cohort Studies, Historical':ab,ti or 'Cohort Study, Historical':ab,ti or 'Historical Cohort Study':ab,ti or 'Study, Historical Cohort':ab,ti or 'Studies, Historical Cohort':ab,ti or 'Incidence Studies':ab,ti or 'Incidence Study':ab,ti or 'Studies, Incidence':ab,ti or 'Study, Incidence':ab,ti or 'Cohort Studies':ab,ti |
| S12 | 10 OR 11 |
| S13 | 3 AND 6 AND 9 AND 12 AND [2010-2022]/py |
| [Exp] is abbreviation of “exploding”, meaning that we investigated studies that registered not only the corresponding term itself, but also the lower MeSH or EMTREE terms than that term. | |
| [ab,ti] means search in the title or abstract | |

MEDLINE search

| S1 | [MESH terms related to physical activity]  Exercise[MeSH] |
| --- | --- |
| S2 | [entry words related to physical activity]  (Physical Activity[Title/Abstract])) OR (Activities, Physical[Title/Abstract])) OR (Activity, Physical[Title/Abstract])) OR (Physical Activities[Title/Abstract])) OR (Exercise, Physical[Title/Abstract])) OR (Exercises, Physical[Title/Abstract])) OR (Physical Exercise[Title/Abstract])) OR (Physical Exercises[Title/Abstract])) OR (Acute Exercise[Title/Abstract])) OR (Acute Exercises[Title/Abstract])) OR (Exercise, Acute[Title/Abstract])) OR (Exercises, Acute[Title/Abstract])) OR (Exercise, Isometric[Title/Abstract])) OR (Exercises, Isometric[Title/Abstract])) OR (Isometric Exercises[Title/Abstract])) OR (Isometric Exercise[Title/Abstract])) OR (Exercise, Aerobic[Title/Abstract])) OR (Aerobic Exercise[Title/Abstract])) OR (Aerobic Exercises[Title/Abstract])) OR (Exercises, Aerobic[Title/Abstract])) OR (Exercise Training[Title/Abstract])) OR (Exercise Trainings[Title/Abstract])) OR (Training, Exercise[Title/Abstract])) OR (Trainings, Exercise[Title/Abstract]) |
| S3 | 1 OR 2 |
| S4 | [MESH terms related to diabetes mellitus]  Diabetes Mellitus [Mesh] |
| S5 | [entry words related to diabetes mellitus]  Diabetes[Title/Abstract] |
| S6 | 4 OR 5 |
| S7 | [MESH terms related to Cardiovascular Diseases]  Cardiovascular Diseases [Mesh] |
| S8 | [entry words related to Cardiovascular Diseases]  (Disease, Cardiovascular[Title/Abstract]) OR (Cardiovascular Disease[Title/Abstract]) OR (Major Adverse Cardiac Events[Title/Abstract]) OR (Cardiac Events[Title/Abstract]) OR (Cardiac Event[Title/Abstract]) OR (Event, Cardiac[Title/Abstract]) OR (Adverse Cardiac Event[Title/Abstract]) OR (Adverse Cardiac Events[Title/Abstract]) OR (Cardiac Event, Adverse[Title/Abstract]) OR (Cardiac Events, Adverse[Title/Abstract]) OR (CVD[Title/Abstract]) |
| S9 | 7 OR 8 |
| S10 | [MESH terms related to Cohort Studies]  Cohort Studies[MeSH] |
| S11 | [entry words related to Cohort Studies]  (longitudinal[Title/Abstract]) OR (prospective[Title/Abstract]) OR (cohort[Title/Abstract]) OR (follow up[Title/Abstract]) OR (Cohort Study[Title/Abstract]) OR (Studies, Cohort[Title/Abstract]) OR (Study, Cohort[Title/Abstract]) OR (Concurrent Studies[Title/Abstract]) OR (Studies, Concurrent[Title/Abstract]) OR (Concurrent Study[Title/Abstract]) OR (Study, Concurrent[Title/Abstract]) OR (Closed Cohort Studies[Title/Abstract]) OR (Cohort Studies, Closed[Title/Abstract]) OR (Closed Cohort Study[Title/Abstract]) OR (Cohort Study, Closed[Title/Abstract]) OR (Study, Closed Cohort[Title/Abstract]) OR (Studies, Closed Cohort[Title/Abstract]) OR (Birth Cohort Studies[Title/Abstract]) OR (Birth Cohort Study[Title/Abstract]) OR (Cohort Studies, Birth[Title/Abstract]) OR (Cohort Study, Birth[Title/Abstract]) OR (Studies, Birth Cohort[Title/Abstract]) OR (Study, Birth Cohort[Title/Abstract]) OR (Analysis, Cohort[Title/Abstract]) OR (Analyses, Cohort[Title/Abstract]) OR (Cohort Analyses[Title/Abstract]) OR (Cohort Analysis[Title/Abstract]) OR (Historical Cohort Studies[Title/Abstract]) OR (Cohort Studies, Historical[Title/Abstract]) OR (Cohort Study, Historical[Title/Abstract]) OR (Historical Cohort Study[Title/Abstract]) OR (Study, Historical Cohort[Title/Abstract]) OR (Studies, Historical Cohort[Title/Abstract]) OR (Incidence Studies[Title/Abstract]) OR (Incidence Study[Title/Abstract]) OR (Studies, Incidence[Title/Abstract]) OR (Study, Incidence[Title/Abstract]) |
| S12 | 10 OR 11 |
| S13 | 3 AND 6 AND 9 AND 12 AND (2010:2022[pdat]) |

Web of Science search

| #1 | [words related to diabetes mellitus]  TS=(Diabetes Mellitus OR Diabetes) |
| --- | --- |
| #2 | [words related to Cardiovascular Diseases ]  TS=(Cardiovascular Diseases OR Cardiovascular Disease OR Disease, Cardiovascular OR Major Adverse Cardiac Events OR Cardiac Events OR Cardiac Event OR Event, Cardiac OR Adverse Cardiac Event OR Adverse Cardiac Events OR Cardiac Event, Adverse OR Cardiac Events, Adverse) |
| #3 | [words related to physical activity ]  TS=(Exercise OR Exercises OR Physical Activity OR Activities, Physical OR Activity, Physical OR Physical Activities OR Exercise, Physical OR Exercises, Physical OR Physical Exercise OR Physical Exercises OR Acute Exercise OR Acute Exercises OR Exercise, Acute OR Exercises, Acute OR Exercise, Isometric OR Exercises, Isometric OR Isometric Exercises OR Isometric Exercise OR Exercise, Aerobic OR Aerobic Exercise OR Aerobic Exercises OR Exercises, Aerobic OR Exercise Training OR Exercise Trainings OR Training, Exercise OR Trainings, Exercise) |
| #4 | [words related to cohort study]  TS=(Cohort Studies OR longitudinal OR prospective OR cohort OR follow up OR Cohort Study OR Studies, Cohort OR Study, Cohort OR Concurrent Studies OR Studies, Concurrent OR Concurrent Study OR Study, Concurrent OR Closed Cohort Studies OR Cohort Studies, Closed OR Closed Cohort Study OR Cohort Study, Closed OR Study, Closed Cohort OR Studies, Closed Cohort OR Birth Cohort Studies OR Birth Cohort Study OR Cohort Studies, Birth OR Cohort Study, Birth OR Studies, Birth Cohort OR Study, Birth Cohort OR Analysis, Cohort OR Analyses, Cohort) |
| #5 | Timespan: 2010-01-01 to 2023-01-01 (Publication Date) |
| #6 | 1 AND 2 AND 3 AND 4 AND 5 |

**Supplementary Table 2.** Information of excluded studies after full-text reading.

| References | Titles of the articles | Reasons for exclusion (after full-text reading) |
| --- | --- | --- |
| [26] | Changes in physical activity and modelled cardiovascular risk following diagnosis of diabetes: 1-year results from the ADDITION-Cambridge trial cohort | The outcome indicator is dietary behavior, which is inconsistent. |
| [36] | Low physical activity is associated with increased arterial stiffness in patients recently diagnosed with type 2 diabetes | The study is a cross-sectional study, the study type does not match. |
| [37] | Four weeks of high-intensity interval training (HIIT) improve the cardiometabolic risk profile of overweight patients with type 1 diabetes mellitus (T1DM) | The outcome indicator is cardiac metabolism, which is inconsistent. |
| [38] | Physical activity, sedentary behavior and all-cause mortality among blacks and whites with diabetes | The outcome indicator is all-cause mortality, which is inconsistent. |
| [39] | Three months monitored metabolic fitness modulates cardiovascular risk factors in diabetic patients | The outcome indicator is cardiovascular risk factors, which is inconsistent. |
| [40] | Changes in behaviors after diagnosis of type 2 diabetes and 10-year incidence of cardiovascular disease and mortality | Physical activity (PA) is not quantified; it is only the change from baseline. |
| [41] | Changes in diet, cardiovascular risk factors and modelled cardiovascular risk following diagnosis of diabetes: 1-year results from the ADDITION-Cambridge trial cohort | The outcome indicator is dietary behavior, which is inconsistent. |

**Supplementary Table 3.** Details of confounding factors considered in each included study

| Study source | confounders |  |  |  |  |  |  |  |  |
| --- | --- | --- | --- | --- | --- | --- | --- | --- | --- |
|  | age, gender | smoking | alcohol | OB | HT | HL | Diet | past CVD | *social |
| Tikkanen-Dolenc H 2017 | Yes | Yes | No | Yes | No | Yes | No | No | No |
| Yerramalla MS 2020 | Yes | Yes | Yes | Yes | Yes | Yes | Yes | Yes | Yes |
| Vepsäläinen T 2010 | Yes | Yes | Yes | Yes | No | Yes | No | No | No |
| Tielemans AJ 2013 | Yes | Yes | Yes | Yes | No | Yes | No | No | No |
| Zethelius B 2014 | Yes | Yes | No | Yes | Yes | Yes | No | No | No |
| Blomster JI 2013 | Yes | Yes | Yes | Yes | Yes | Yes | No | Yes | Yes |
| Enguita-Germán M 2021 | Yes | Yes | Yes | Yes | Yes | No | No | Yes | Yes |
| Sone H 2013 | Yes | Yes | No | Yes | Yes | Yes | Yes | No | No |
| Yung-Feng Yen 2022 | Yes | Yes | Yes | Yes | Yes | Yes | Yes | No | Yes |
| Ruth E. Brown 2014 | Yes | Yes | No | Yes | Yes | No | No | Yes | Yes |
| Yijia Chen 2022 | Yes | Yes | Yes | Yes | No | No | Yes | No | Yes |
| Kosuke Inoue 2020 | Yes | Yes | Yes | Yes | Yes | Yes | No | No | Yes |

**Supplem Abbreviations**: OB, obesity or body mass index; HT, hypertension or blood pressure; HL, hyperlipidemia or total/LDL cholesterol value

*social background such as economic status, educational level, marital status etc.

#### Supplementary Table 4. Criteria for assessment of bias / study quality

| **1st Author** | **Year** | **Representativeness of cohort** | **Selection of non-exposed** | **Ascertainment of exposure** | **Demonstration that outcome of interest was not present at start of stud y** | **Comparability of cases and controls on the basis of the design or analysis** | **Loss to follow-up** | **follow-up long enough for outcomes to occur** | **Assessment of outcome** | **Quality score** |
| --- | --- | --- | --- | --- | --- | --- | --- | --- | --- | --- |
| Tikkanen-Dolenc H | 2017 | 1 | 1 | 1 | 1 | 2 | 0 | 1 | 1 | 8 |
| Yerramalla MS | 2020 | 0 | 1 | 1 | 1 | 2 | 0 | 0 | 1 | 6 |
| Vepsäläinen T | 2010 | 1 | 1 | 1 | 1 | 2 | 0 | 1 | 1 | 8 |
| Tielemans AJ | 2013 | 1 | 1 | 0 | 1 | 2 | 1 | 0 | 1 | 7 |
| Zethelius B | 2014 | 1 | 1 | 0 | 1 | 2 | 0 | 0 | 1 | 6 |
| Blomster JI | 2013 | 1 | 1 | 0 | 1 | 2 | 0 | 0 | 1 | 6 |
| Enguita-Germán M | 2021 | 1 | 1 | 0 | 1 | 2 | 1 | 0 | 1 | 7 |
| Sone H | 2013 | 1 | 1 | 0 | 1 | 2 | 0 | 0 | 1 | 6 |
| Yung-Feng Yen | 2022 | 1 | 1 | 0 | 1 | 2 | 0 | 1 | 1 | 7 |
| Ruth E. Brown | 2014 | 1 | 1 | 0 | 1 | 2 | 0 | 0 | 1 | 6 |
| Yijia Chen | 2022 | 1 | 1 | 0 | 1 | 2 | 0 | 0 | 1 | 6 |
| Kosuke Inoue | 2010 | 1 | 1 | 0 | 1 | 2 | 0 | 0 | 1 | 6 |

- - *Representativeness of cohort (0 = no description or selected group of users (e.g. nurses), 1 = otherwise)*
  - *Selection of non-exposed (0 = no description or drawn from different source than exposed, 1= otherwise)*
  - *Ascertainment of exposure (0 = no description or non-validated self report, 1 = otherwise)*
  - *Demonstration that outcome of interest was not present at start of study (0 =no, 1 =yes)*
  - *Comparability of cohorts on the basis of the design or analysis (0 = otherwise, 1 = study controls for any additional factor)*
  - *Loss to follow-up (0 = no description or greater than 5%, 1 = otherwise)*
  - *follow-up long enough for outcomes to occur (0 = no, 1 = yes)*
  - *Assessment of outcome (0 = no description or incidence measured by self report of doctor diagnosis, 1 = otherwise)*

| **outcomes** | **study** | **RR** | **LCI** | **UCI** | **lnRR** | **lnLCI** | **lnUCI** |
| --- | --- | --- | --- | --- | --- | --- | --- |
| CVD events | Tikkanen-Dolenc H 2017 | 0.63 | 0.41 | 0.97 | -0.46 | -0.90 | -0.04 |
| CVD mortality | Yerramalla MS 2020 | 0.40 | 0.16 | 0.96 | -0.92 | -1.83 | -0.04 |
| CVD mortality | Vepsäläinen T 2010 | 0.63 | 0.45 | 0.88 | -0.46 | -0.80 | -0.13 |
| total CVD events | Tielemans AJ 2013 | 0.97 | 0.69 | 1.37 | -0.03 | -0.37 | 0.31 |
| Fatal/nonfatal CVD | Zethelius B 2014 | 0.39 | 0.30 | 0.50 | -0.94 | -1.19 | -0.69 |
| CVD events | Blomster JI 2013 | 0.81 | 0.68 | 0.97 | -0.21 | -0.39 | -0.03 |
| CVD events | Enguita-Germán M 2021 | 0.72 | 0.61 | 0.84 | -0.33 | -0.49 | -0.17 |
| CHD | Sone H 2013 | 0.77 | 0.43 | 1.38 | -0.26 | -0.84 | 0.32 |
| CVD mortality | Yung-Feng Yen 2022 | 0.78 | 0.55 | 1.13 | -0.25 | -0.60 | 0.12 |
| CVD mortality | Ruth E. Brown 2014 | 0.59 | 0.37 | 0.95 | -0.53 | -1.00 | -0.05 |
| CVD mortality | Yijia Chen 2022 | 0.46 | 0.33 | 0.64 | -0.78 | -1.11 | -0.45 |
| CVD events | Kosuke Inoue 2020 | 0.53 | 0.39 | 0.67 | -0.63 | -0.94 | -0.40 |

#### Supplementary Table 5. Information on data included in the study

| Study | Frequency | Duration | Intensity | Original information | Assigned MET h/wk |
| --- | --- | --- | --- | --- | --- |
| Tikkanen-Dolenc H 2017 |  |  |  | Sedentary 0-10METh/w | 5METh/w |
|  |  |  |  | Moderately active 10-40METh/w | 25METh/w |
|  |  |  |  | Active ＞40METh/w | 55METh/w |
| Yerramalla MS 2020 |  | 0 |  | inactive | 0METh/w |
|  |  | 0.625h | MPA=3-5.9MET（4.5） | below recommendation（＜1.25h/w VPA ） | 4.69METh/w |
|  |  | 1.875h | VPA=≥6MET（7.5） | follow recommendation（＞1.25h/w VPA ） | 14.06METh/w |
| Tielemans AJ 2013 |  | 45min/session | MPA=3-5.9MET（4.5） | None or mild PA once a week or more | 0METh/w |
|  |  |  | VPA=≥6MET（7.5） | Moderate or vigorous PA once a week or more | 11.25 METh/w |
| Zethelius B 2014 | 1 | 45min/session | 4.5 MET | low activity both at baseline and during follow up | 3.38METh/w |
|  | 3 |  | 4.5 MET | with low activity at baseline and higher during follow-up, or baseline higher activity | 10.13METh/w |
|  | 5 |  | 4.5 MET smith2016 | low activity at baseline and higher during follow up | 16.88METh/w |
| Blomster JI 2013 |  | 45min/session | sedentary behavior 1.0-1.5 MET | sedentary | 0.94METh/w |
|  |  |  | LPA1.6-2.9 MET | mild | 1.69METh/w |
|  |  |  | MVPA=6MET | moderate and vigorous | 4.5METh/w |
| Sone H 2013 |  |  |  | ≤3.7METh/w | 1.85METh/w |
|  |  |  |  | 3.8-15.3METh/w | 9.55METh/w |
|  |  |  |  | ≥15.4METh/w | 21.15METh/w |
| Yung-Feng Yen 2022 |  |  |  | 0 | 0METh/w |
|  |  |  |  | 1-800METm/w | 6.67METh/w |
|  |  |  |  | ＞800METm/w | 13.33METh/w |
| Yijia Chen 2022 |  |  |  | 1.1METh/d | 7.7METh/w |
|  |  |  |  | 5.3METh/d | 37.1METh/w |
|  |  |  |  | 12.0METh/d | 84.0METh/w |
|  |  |  |  | 24.0METh/d | 168METh/w |
| Kosuke Inoue 2020 |  |  |  | <20 METh/w | 10METh/w |
|  |  |  |  | 20-97 METsh/w | 58.5METh/w |
|  |  |  |  | ≥97.5 METsh/w | 136METh/w |

**Supplementary Table 6. Summary of MET h/week dose assignment calculations for the cohort studies included in the dose–response meta-analysis**

**Supplementary Table 7** Specific data on the dose-response relationship between PA and CVD mortality.

|  | study | cat | dose | rr | l | u | cases | peryears | type | obs |
| --- | --- | --- | --- | --- | --- | --- | --- | --- | --- | --- |
| 1 | Yerramalla MS 2020 | 1 | 0 | 1 | 1 | 1 | 15 | 1557.6 | 1 | 1 |
|  |  | 2 | 4.69 | 0.84 | 0 | 1 | 28 | 3546.4 | 1 | 2 |
|  |  | 3 | 14.06 | 0.4 | 0 | 1 | 12 | 3924.8 | 1 | 3 |
| 2 | Zethelius B 2014 | 1 | 3.375 | 1 | 1 | 1 | 64 | 19996.8 | 1 | 4 |
|  |  | 2 | 10.13 | 0.47 | 0 | 1 | 64 | 54220.8 | 1 | 5 |
|  |  | 3 | 16.88 | 0.31 | 0 | 1 | 12 | 13425.6 | 1 | 6 |
| 3 | Yung-Feng Yen 2022 | 1 | 0 | 1 | 1 | 1 | 108 | 17980 | 1 | 7 |
|  |  | 2 | 6.67 | 0.54 | 0 | 1 | 48 | 13470 | 1 | 8 |
|  |  | 3 | 13.33 | 0.78 | 1 | 1 | 83 | 17140 | 1 | 9 |
| 4 | Yijia Chen 2022 | 1 | 1.1 | 1 | 1 | 1 | 181 | 21253.5 | 1 | 10 |
|  |  | 2 | 5.3 | 0.86 | 1 | 1 | 144 | 24187.5 | 1 | 11 |
|  |  | 3 | 12 | 0.53 | 0 | 1 | 54 | 16605 | 1 | 12 |
|  |  | 4 | 24 | 0.46 | 0 | 1 | 47 | 19863 | 1 | 13 |
| 5 | Kosuke Inoue 2020 | 1 | 10 | 1 | 1 | 1 | 46 | 3352 | 1 | 14 |
|  |  | 2 | 58.5 | 0.93 | 1 | 1 | 111 | 6704 | 1 | 15 |
|  |  | 3 | 136 | 0.49 | 0 | 1 | 106 | 3352 | 1 | 16 |

**Supplementary Table 8.** Specific data on the dose–response relationship between PA and CVD events.

| study |  | cat | dose | rr | l | u | cases | peryears | type | obs |
| --- | --- | --- | --- | --- | --- | --- | --- | --- | --- | --- |
| 1 | **Tikkanen-Dolenc H 2017** | 1 | 5 | 1 | 1 | 1 | 68 | 7096.7 | 1 | 1 |
|  | CVD | 2 | 25 | 0.66 | 0.43 | 1.01 | 68 | 10351.5 | 1 | 2 |
|  |  | 3 | 55 | 0.63 | 0.41 | 0.97 | 68 | 3914 | 1 | 3 |
| 2 | **Yerramalla MS 2020** | 1 | 0 | 1 | 1 | 1 | 15 | 1557.6 | 1 | 4 |
|  | CVD-fatal | 2 | 4.69 | 0.84 | 0.41 | 1.7 | 28 | 3546.4 | 1 | 5 |
|  |  | 3 | 14.06 | 0.4 | 0.16 | 0.96 | 12 | 3924.8 | 1 | 6 |
| 3 | **Tielemans AJ 2013** | 1 | 0 | 1 | 1 | 1 | 58 | 5328 | 1 | 7 |
|  | CVD total | 2 | 11.25 | 0.97 | 0.69 | 1.37 | 87 | 9803.9 | 1 | 8 |
| 4 | **Zethelius B 2014** | 1 | 3.375 | 1 | 1 | 1 | 313 | 19996.8 | 1 | 9 |
|  | Fatal/nonfatal CVD | 2 | 10.125 | 0.6 | 0.51 | 0.69 | 447 | 54220.8 | 1 | 10 |
|  |  | 3 | 16.875 | 0.39 | 0.3 | 0.5 | 57 | 13425.6 | 1 | 11 |
| 5 | **Blomster JI 2013** | 1 | 0.9375 | 1 | 1 | 1 | 382 | 8045 | 1 | 12 |
|  | CVD | 2 | 1.6875 | 1.06 | 0.9 | 1.26 | 382 | 22090 | 1 | 13 |
|  |  | 3 | 4.5 | 0.81 | 0.68 | 0.97 | 382 | 25565 | 1 | 14 |
| 6 | **Sone H 2013** | 1 | 1.85 | 1 | 1 | 1 | 71 | 4435.55 | 1 | 15 |
|  | CHD or stroke | 2 | 9.55 | 0.96 | 0.61 | 1.5 | 75 | 4741.45 | 1 | 16 |
|  |  | 3 | 21.15 | 0.68 | 0.42 | 1.11 | 57 | 4524.1 | 1 | 17 |
| 7 | **Yung-Feng Yen 2022** | 1 | 0 | 1 | 1 | 1 | 108 | 17980 | 1 | 18 |
|  | CVD-fatal | 2 | 6.67 | 0.54 | 0.36 | 0.84 | 48 | 13470 | 1 | 19 |
|  |  | 3 | 13.33 | 0.78 | 0.55 | 1.13 | 83 | 17140 | 1 | 20 |
| 8 | **Yijia Chen 2022** | 1 | 1.1 | 1 | 1 | 1 | 181 | 21253.5 | 1 | 21 |
|  | CVD-fatal | 2 | 5.3 | 0.86 | 0.69 | 1.08 | 144 | 24187.5 | 1 | 22 |
|  |  | 3 | 12 | 0.53 | 0.38 | 0.72 | 54 | 16605 | 1 | 23 |
|  |  | 4 | 24 | 0.46 | 0.33 | 0.64 | 47 | 19863 | 1 | 24 |
| 9 | **Kosuke Inoue 2020** | 1 | 10 | 1 | 1 | 1 | 84 | 2352 | 1 | 25 |
|  | nonfatal CVD events | 2 | 58.5 | 0.72 | 0.54 | 0.97 | 192 | 4304 | 1 | 26 |
|  |  | 3 | 136 | 0.59 | 0.42 | 0.85 | 93 | 1856 | 1 | 27 |
|  | Fatal CVD events | 1 | 10 | 1 | 1 | 1 | 46 | 3352 | 1 | 28 |
|  |  | 2 | 58.5 | 0.93 | 0.66 | 1.33 | 111 | 6704 | 1 | 29 |
|  |  | 3 | 136 | 0.49 | 0.34 | 0.7 | 106 | 3352 | 1 | 30 |

**Supplementary Figure 1.** Egger's tests are used to estimate asymmetry of data. The p-value less than 0.05 implicates publication bias. The P value here is equal to 0.286.

**
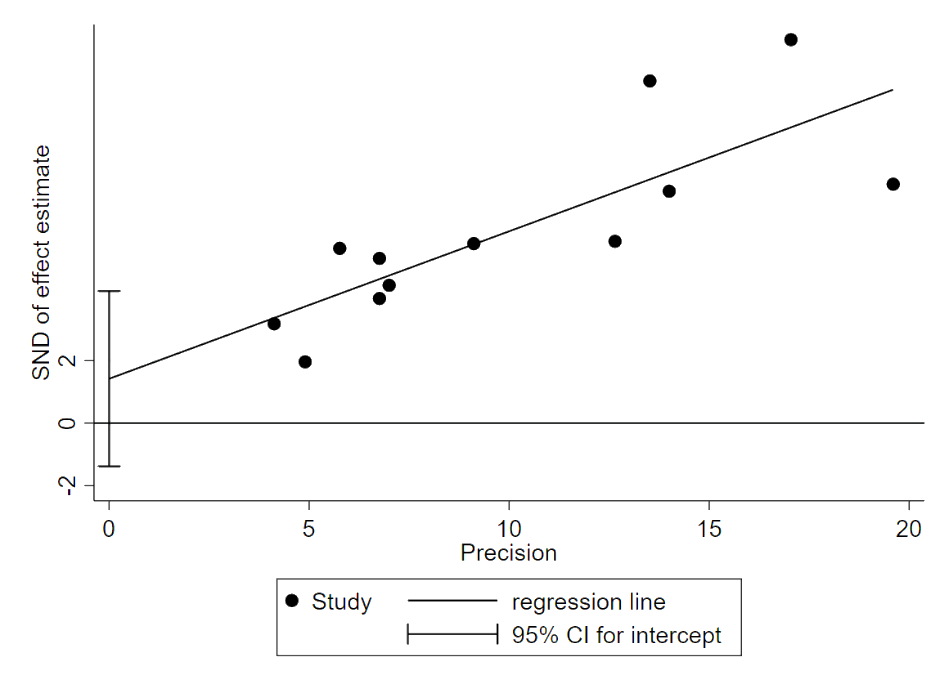
**

**Supplementary Figure 2-10.** Forest plot of study-specific relative risk for CVD incidence

**Supplementary Figure 2.** Forest plot of study-specific relative risk for CVD incidence in follow-up years＞10 years and ≤10 years, respectively.


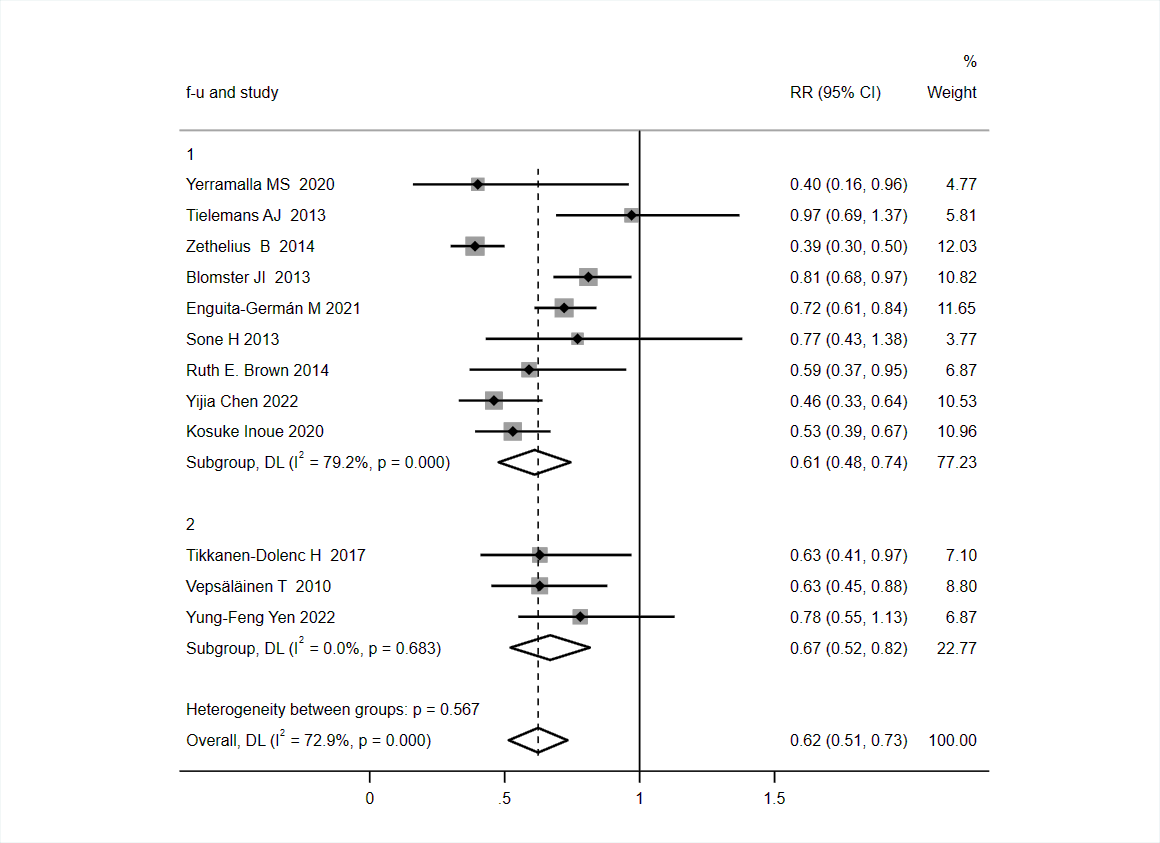


**Supplementary Figure 3**. Forest plot of study-specific relative risk for CVD incidence in type 1 diabetes mellitus and type2 diabetes mellitus, respectively.


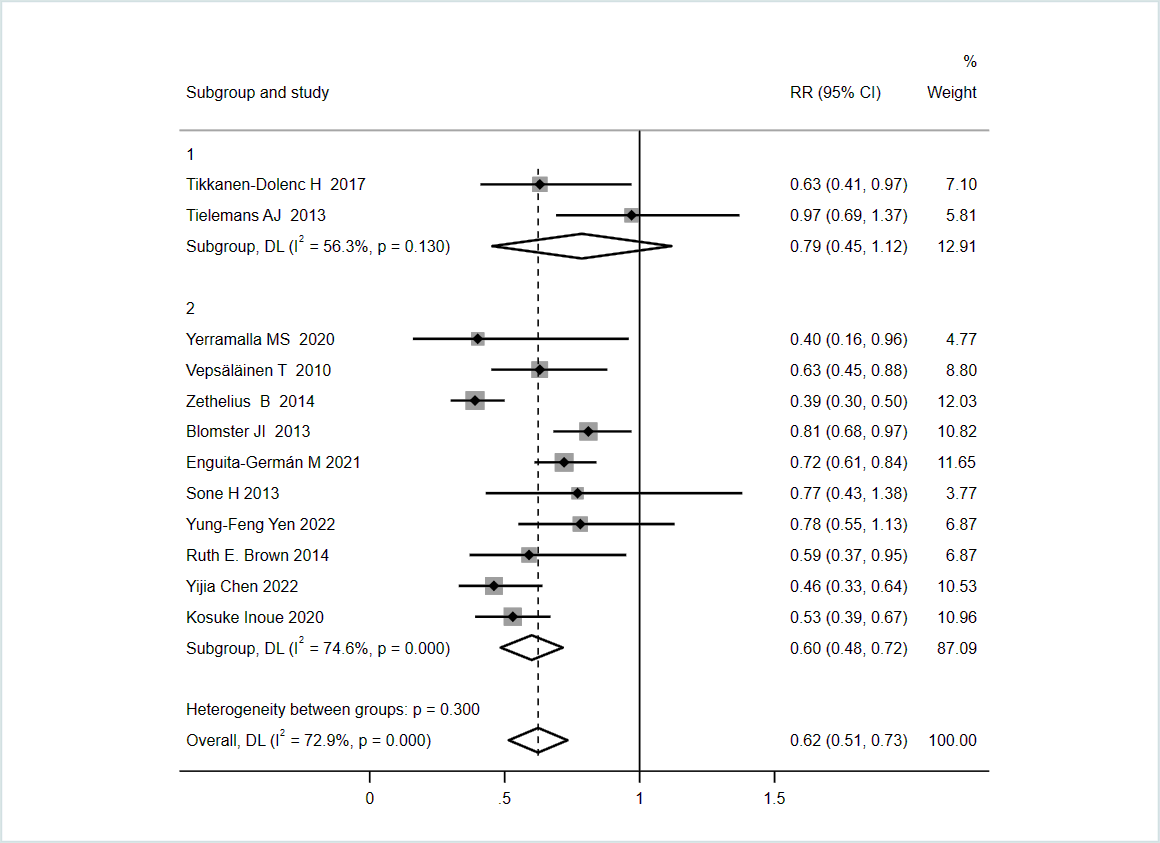


**Supplementary Figure 4.** Forest plot of study-specific relative risk for CVD incidence in duration of diabetes mellitus.


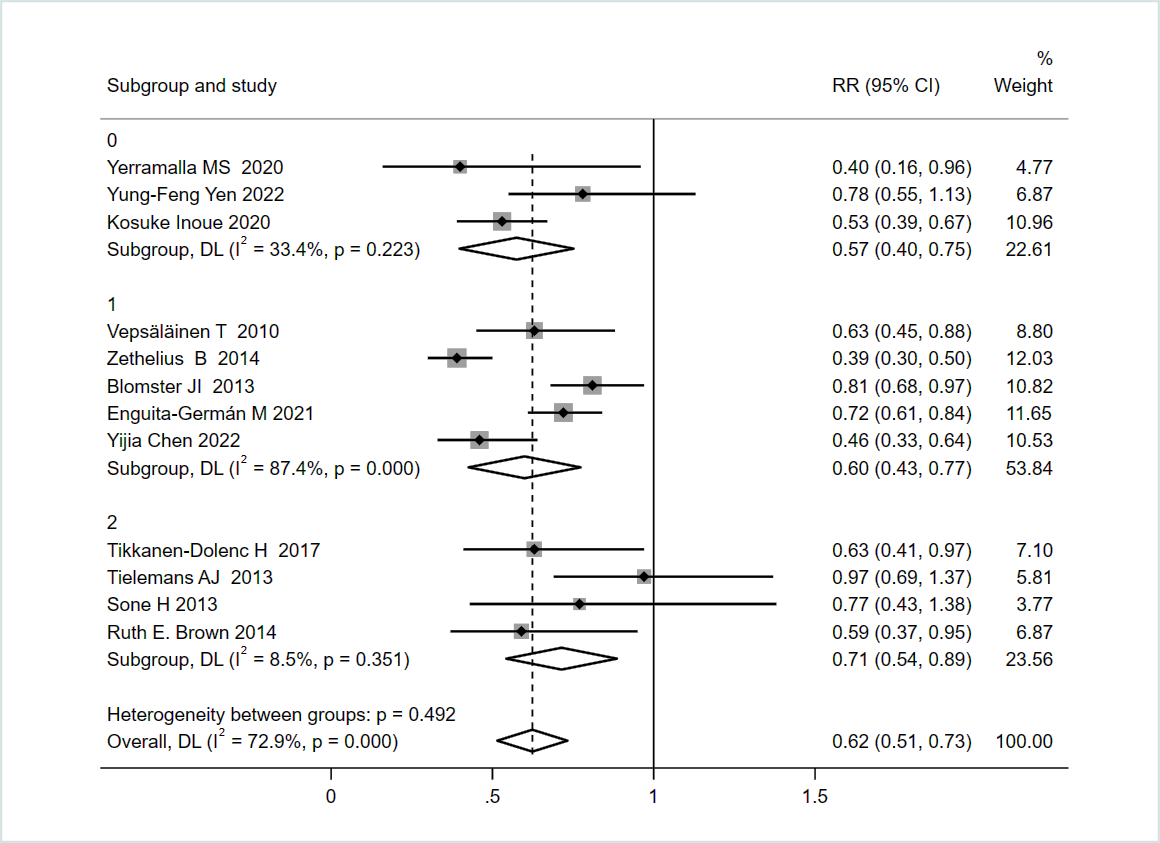


**Supplementary Figure 5.** Forest plot of study-specific relative risk for CVD incidence in age ＜60 years and ≥60 years, respectively.
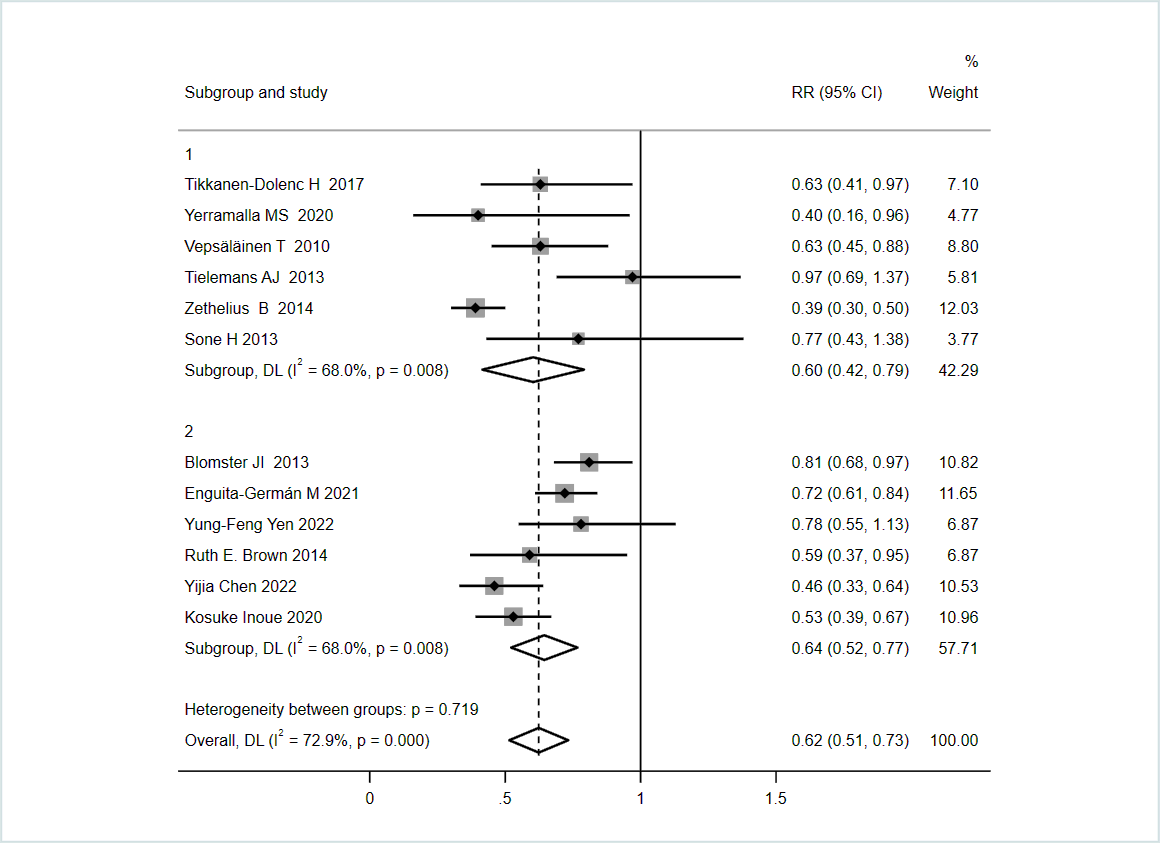


**Supplementary Figure 6**. Forest plot of study-specific relative risk for CVD incidence in LTPA and total PA, respectively.
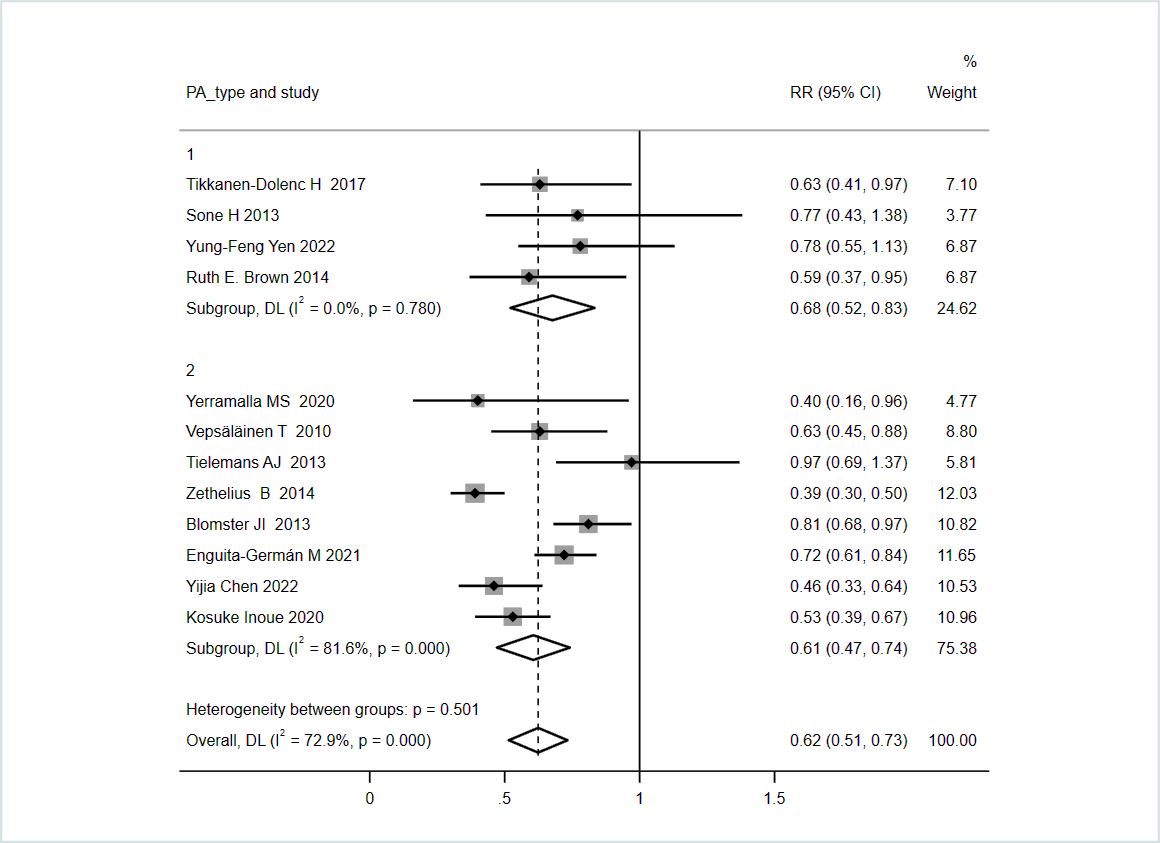


**Supplementary Figure 7**. Forest plot of study-specific relative risk for CVD incidence in man%.


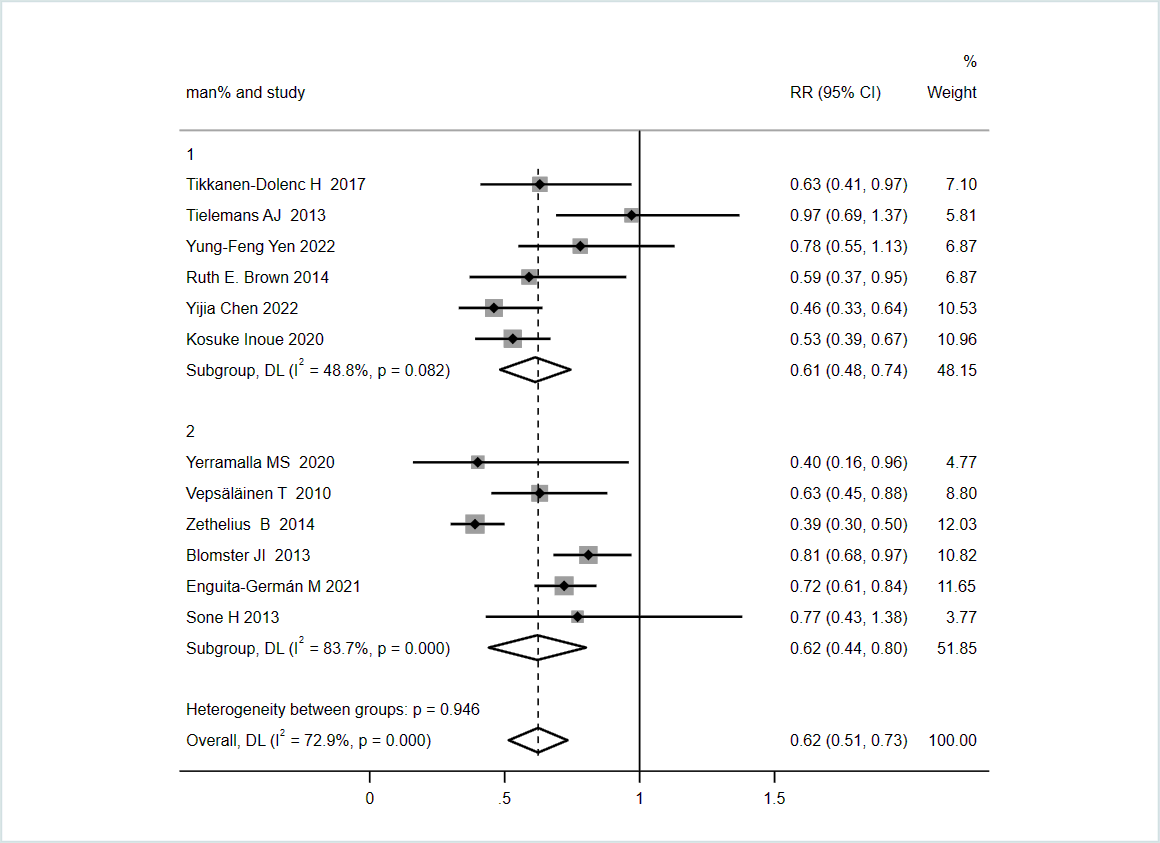


**Supplementary Figure 8**. Forest plot of study-specific relative risk for CVD incidence in validation of PA questionnaire.


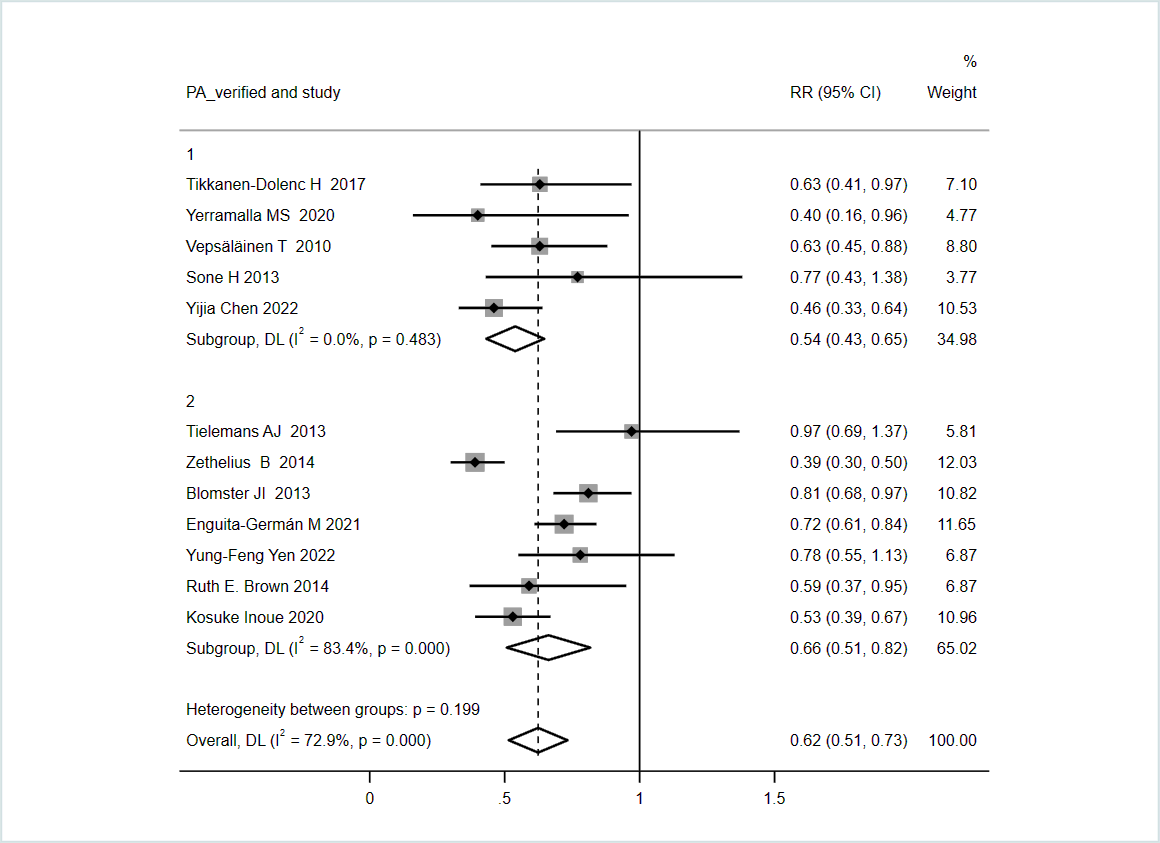


**Supplementary Figure 9**. Forest plot of study-specific relative risk for CVD incidence in Asia, Europe, north America, and Mixtures respectively.

**
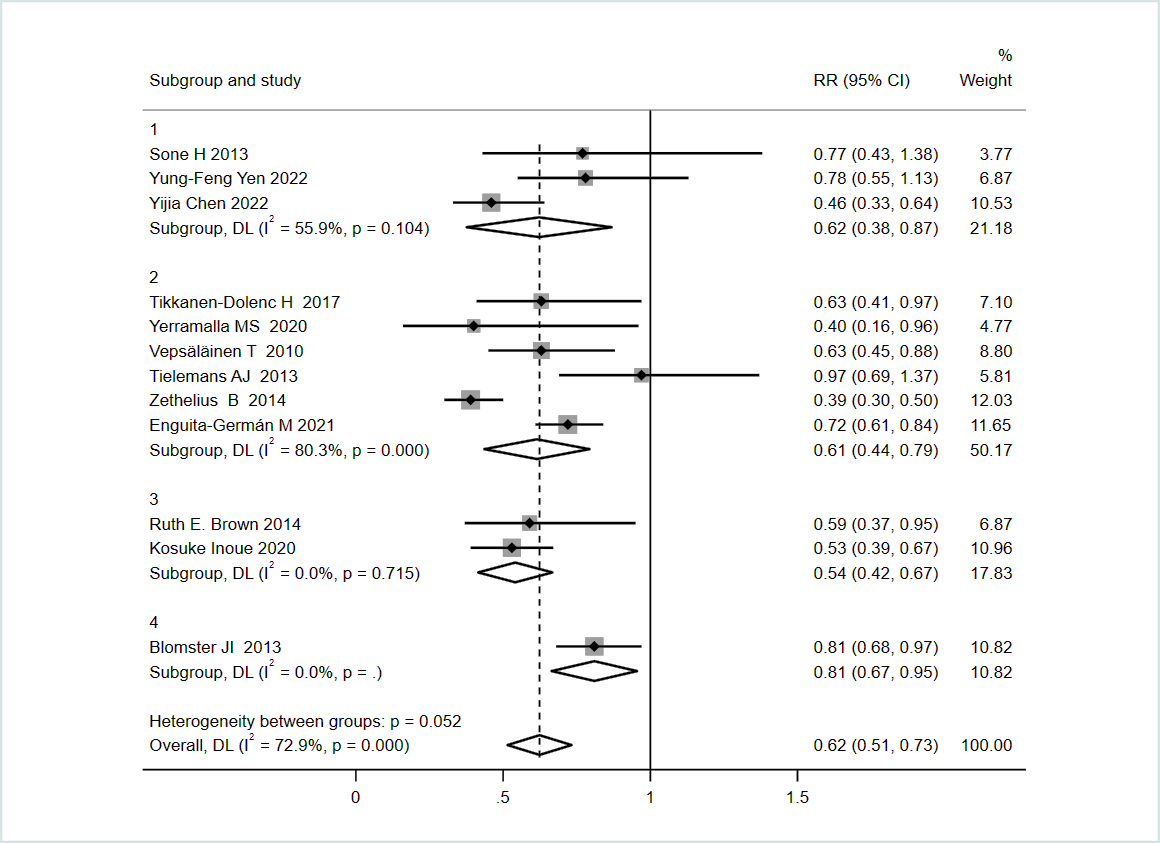
**

**Supplementary Figure 10.** Forest plot of study-specific relative risk for CVD incidence in BMI ≤25 kg/m^2^ , 25＜BMI ＜30 kg/m^2^ and BMI≥30 kg/m^2^, respectively.


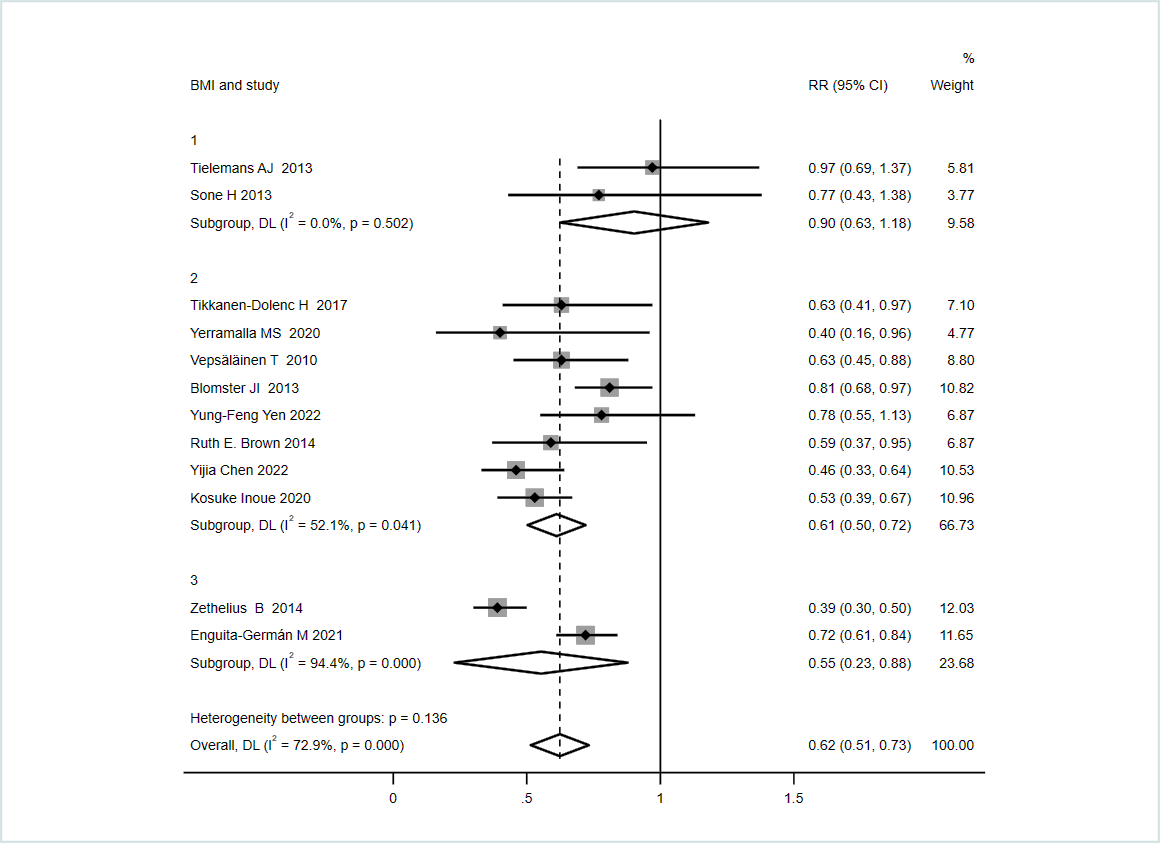


**Supplementary Figure 11. Sensitivity-analysis plot**

1. Barakat A, Williams KM, Prevost AT, Kinmonth AL, Wareham NJ, Griffin SJ, et al. Changes in physical activity and modelled cardiovascular risk following diagnosis of diabetes: 1-year results from the ADDITION-Cambridge trial cohort. Diabet Med 2013 Feb; 30:233-8[doi:10.1111/j.1464-5491.2012.03765.x] [Medline:22913463]
2. Funck KL, Laugesen E, Hoyem P, Fleischer J, Cichosz SL, Christiansen JS, et al. Low Physical Activity Is Associated With Increased Arterial Stiffness in Patients Recently Diagnosed With Type 2 Diabetes. Am J Hypertens 2016 Jul; 29:882-8[doi:10.1093/ajh/hpv197] [Medline:26714500]
3. Minnebeck K, Vorona E, Zinn S, Gellner R, Hinder J, Brand SM, et al. Four weeks of high-intensity interval training (HIIT) improve the cardiometabolic risk profile of overweight patients with type 1 diabetes mellitus (T1DM). Eur J Sport Sci 2021 Aug; 21:1193-203[doi:10.1080/17461391.2020.1810782] [Medline:32790537]
4. Glenn KR, Slaughter JC, Fowke JH, Buchowski MS, Matthews CE, Signorello LB, et al. Physical activity, sedentary behavior and all-cause mortality among blacks and whites with diabetes. Ann Epidemiol 2015 Sep; 25:649-55[doi:10.1016/j.annepidem.2015.04.006] [Medline:26141652]
5. Cirilli I, Silvestri S, Marcheggiani F, Olivieri F, Galeazzi R, Antonicelli R, et al. Three Months Monitored Metabolic Fitness Modulates Cardiovascular Risk Factors in Diabetic Patients. Diabetes Metab J 2019 Dec; 43:893-7[doi:10.4093/dmj.2018.0254] [Medline:31339009]
6. Strelitz J, Ahern AL, Long GH, Boothby CE, Wareham NJ, Griffin SJ. Changes in behaviors after diagnosis of type 2 diabetes and 10-year incidence of cardiovascular disease and mortality. Cardiovasc Diabetol 2019 Aug 1; 18:98[doi:10.1186/s12933-019-0902-5] [Medline:31370851]
7. Savory LA, Griffin SJ, Williams KM, Prevost AT, Kinmonth AL, Wareham NJ, et al. Changes in diet, cardiovascular risk factors and modelled cardiovascular risk following diagnosis of diabetes: 1-year results from the ADDITION-Cambridge trial cohort. Diabet Med 2014 Feb; 31:148-55[doi:10.1111/dme.12316] [Medline:24102972]

**References**
